# Modeling approaches for assessing device-based measures of energy expenditure in school-based studies of body weight status

**DOI:** 10.3389/fams.2024.1399426

**Published:** 2024-06-25

**Authors:** Gilson D. Honvoh, Roger S. Zoh, Anand Gupta, Mark E. Benden, Carmen D. Tekwe

**Affiliations:** 1 Department of Pediatrics, Cincinnati Children’s Hospital Medical Center, Cincinnati, OH, United States; 2 Department of Epidemiology and Biostatistics, Indiana University, Bloomington, IN, United States; 3 Department of Internal Medicine, University of Texas Southwestern Medical Center, Dallas, TX, United States; 4 Department of Environmental and Occupational Health, Texas A&M University, College Station, TX, United States

**Keywords:** B-splines, behavioral intervention, cluster-randomized trial, functional data analysis, physical activity, quantile regression

## Abstract

**Background:**

Obesity has become an important threat to children’s health, with physical and psychological impacts that extend into adulthood. Limited physical activity and sedentary behavior are associated with increased obesity risk. Because children spend approximately 6 h each day in school, researchers increasingly study how obesity is influenced by school-day physical activity and energy expenditure (EE) patterns among school-aged children by using wearable devices that collect data at frequent intervals and generate complex, high-dimensional data. Although clinicians typically define obesity in children as having an age-and sex-adjusted body mass index (BMI) value in the high percentiles, the relationships between school-based physical activity interventions and BMI are analyzed using traditional linear regression models, which are designed to assess the effects of interventions among children with average BMI, limiting insight regarding the effects of interventions among children categorized as overweight or obese.

**Methods:**

We investigate the association between wearable device–based EE measures and age-and sex-adjusted BMI values in data from a cluster-randomized, school-based study. We express and analyze EE levels as both a scalar-valued variable and as a continuous, high-dimensional, functional predictor variable. We investigate the relationship between school-day EE (SDEE) and BMI using four models: a linear mixed-effects model (LMEM), a quantile mixed-effects model (QMEM), a functional mixed-effects model (FMEM), and a functional quantile mixed-effects model (FQMEM). The LMEM and QMEM include SDEE as a summary measure, whereas the FMEM and FQMEM allow for the modeling of SDEE as a high-dimensional covariate. The FMEM and FQMEM allow the influence of the time of day at which physical activity is performed to be assessed, which is not possible using the LMEM or the QMEM. The FMEM assesses how frequently collected SDEE data influences mean BMI, whereas the FQMEM assesses the effects on quantile levels of BMI.

**Results:**

The LMEM and QMEM detected a statistically significant effect of overall mean SDEE on log (BMI) (the natural logarithm of BMI) after adjusting for intervention, age, race, and sex. The FMEM and FQMEM provided evidence for statistically significant associations between SDEE and log (BMI) for only a short time interval. Being a boy or being assigned a stand-biased desk is associated with a lower log (BMI) than being a girl or being assigned a traditional desk. Across our models, age was not a statistically significant covariate, and white students had significantly lower log (BMI) than non-white students in quantile models, but this significant effect was observed for only the 10th and 50th quantile levels of BMI. The functional regression models allow for additional interpretations of the influence of EE patterns on age-and sex-adjusted BMI, whereas the quantile regression models enable the influence of EE patterns to be assessed across the entire BMI distribution.

**Conclusion:**

The FQMEM is recommended when interest lies in assessing how device-monitored SDEE patterns affect children of all body types, as this model is robust and able to assess intervention effects across the full BMI distribution. However, the sample size must be sufficiently large to adequately power determinations of covariate effects across the entire BMI distribution, including the tails.

## 1 Introduction

Approximately 90% of children diagnosed with type 2 diabetes are classified as either overweight or obese (1). Obesity has been linked to various factors, including a chronic imbalance between energy expenditure (EE) and energy intake, environmental exposures, and genetic predispositions. However, the exact contributions of EE to obesity development remain unclear (2). To combat the growing obesity epidemic among children, behavioral researchers increasingly employ targeted, school-based interventions designed to reduce school-day sedentary behaviors among children (3–5). The effects of these interventions on physical activity (PA) are often monitored using wearable devices, such as accelerometers, which collect frequent measures of estimated calorie expenditures or the number of steps taken (6, 7). Wearable devices typically record data at either the second or minute level over multiple days to monitor PA intensity. Often, these measures are used to estimate the metabolic equivalent of tasks, which can be used to derive the amount of time spent performing sedentary, light, moderate, or vigorous PA (8). Alternatively, the data collected over time by wearable devices can be represented by scalar-valued summary numbers such as mean EE or total EE, or by curves (9–12). When data are presented as curves, functional data analysis, which treats curves as the unit of statistical analysis, is a modeling strategy (13–15). Functional data analysis applies data reductions techniques to the curves and subsequently uses regression approaches for statistical modeling. The data reductions can simply consist in summarizing the data from minute-level observations into hourly mean EE values (11). Furthermore, more complex statistical data reduction techniques, such as functional principal components analyses or polynomial basis expansions, have also been used to for approximating the mean of the curves data have also been used (9, 10, 13, 16–19). Polynomial basis expansions approximate curves by describing their shapes using a few key features, summarizing the information contained within curves into basis functions that adequately capture patterns. Unlike summary statistics, such as the mean, which account for only one source of variation in the data, each basis function accounts for a different source of variation (10). Parametric regression approaches, such as nonlinear or polynomial mixed-effects models, have been considered in functional data settings to parametrically model the effects of curves on an outcome (20–22); however, these approaches are limited by the requirement for strong assumptions regarding the shape of the curve. Thus, semiparametric and nonparametric approaches, which provide more flexibility for fitting curves to data by not requiring a specific parametric form, are standard approaches for analyzing functional data (23–25). Additionally, the ability of these approaches to easily accommodate the high dimensionality of functional data is desirable (13, 14).

In children, overweight and obesity are defined according to age-and sex-adjusted body mass index (BMI) values in the upper ranges (26, 27). However, most studies assessing the impacts of behavioral interventions on BMI rely on traditional linear regression models, which are designed to assess the effects of intervention among children within the *normal* BMI range and have limited ability to assess the effects of interventions among children classified as overweight or obese. Thus, statistical approaches that permit the evaluation of covariate effects across the entire BMI distribution are preferable when assessing the effects of interventions among children classified as overweight or obese (28). Quantile regression is a statistical technique used to estimate the effects of predictors on quantile functions of a response variable (28–33), such as the median (50th), 85th, or 95th quantiles. Quantile regression is advantageous compared with linear regression because quantile regression does not require the regression residuals to be normally distributed. Using classical mean regression models, such as linear regression, to model BMI as an outcome can provide incomplete information regarding BMI values that lie beyond the mean value, such as values within the distribution tails. Additionally, covariates such as PA and age may have differential impacts on different quantile levels. Therefore, statistical approaches that allow covariate effects to be assessed across the full spectrum of quantile functions are preferable when using BMI as an outcome in obesity studies (28, 29).

Different approaches have been used for assessing the relationship between device-based measures of PA patterns and BMI. Wendel et al. (34) recently used classical linear regression to analyze the effects of introducing stand-biased desks in school on the average change in BMI. Their results indicated that compared with using conventional desks, using stand-biased desks significantly reduced the average change in BMI (*p* = 0.04). However, analyzing the average change in BMI does not allow for the assessment of how standing desk use affects values above or below the average BMI change. Benden et al. (3) reported that children who used stand-biased desks had a significantly higher mean EE (estimate [Est.] = 0.16, standard error [SE] = 0.04, *p* < 0.001) than students who used traditional desks. Benden et al. used a hierarchical LMEM to assess the impacts of standing desk use on average SDEE (as a measure of PA); however, this approach is limited to assessing the impacts of standing desk use for those children with “average” SDEE values and cannot assess impacts for those children with SDEE values above or below the average. Additionally, the hierarchical LMEM employed did not model SDEE data as curves.

Trinh et al. (35) studied the effects of PA patterns at baseline on the 3-year change in BMI among elementary school–aged children in Australia and found little evidence to indicate that baseline PA patterns were predictive of future obesity risk when applying classical regression methods that treated objective measures of PA as a summary statistic (average step count per minute) (35). However, using summary statistics to describe PA intensity does not account for potential diurnal PA patterns (9, 11, 12, 36, 37). Approaches that allow assessments of diurnal patterns in PA have also been considered in the literature. For example, Tekwe et al. (9) used functional principal components methods and scalar-on-function regression to analyze EE data (38). These approaches allowed the impacts of diurnal PA patterns on obesity-related outcomes to be assessed. Augustin et al. (12) also considered semiparametric approaches to describe PA patterns and used a histogram of the PA distribution as a predictor in their regression model. Using data from the Avon Longitudinal Study of Parents and Children, Augustin et al. established that their approach provided better fits than summary statistics–based methods.

Our current work was motivated by the stand-biased desk study in which a school-based PA intervention study was assessed (3). The cluster-randomized study was conducted from 2011 to 2013 in three elementary schools within the College Station Independent School District (CSISD) (3). The study is described in detail elsewhere (3); briefly, at the beginning of the 2011–2012 academic year, 24 teachers from three elementary schools were recruited and randomly assigned to the use of either stand-biased desks with stools [Stand2learn LLC College Station, TX, USA, stand-biased desk (model S2LK04) and stool (model S2LS04)] or traditional desks (model 2200 FBBK Series by Scholar Craft Products, Birmingham, AL) with chairs (9000 Classic Series, by Virco Inc., Torrance, CA, USA) for in-class activities (3). A total of 374 students in second through fourth grades were included in the study at baseline. To calculate BMI, each student’s height and weight were measured at the start of each semester by trained research assistants. Study participants were required to wear calibrated BodyMedia SenseWear^®^ armband devices (BodyMedia, Pittsburgh, PA) during school hours for 1 school week in each semester from Fall 2011 to Spring 2013. The devices recorded subject-specific step counts and caloric EE per minute while worn. All study participants consented or assented to participate in the study, and consent to participate was obtained from the parents or legal guardians of all participants. The study protocol was approved by the Institutional Review Board, Human Subjects Program at Texas A&M and the CSISD Review Board.

**FIGURE 1:**
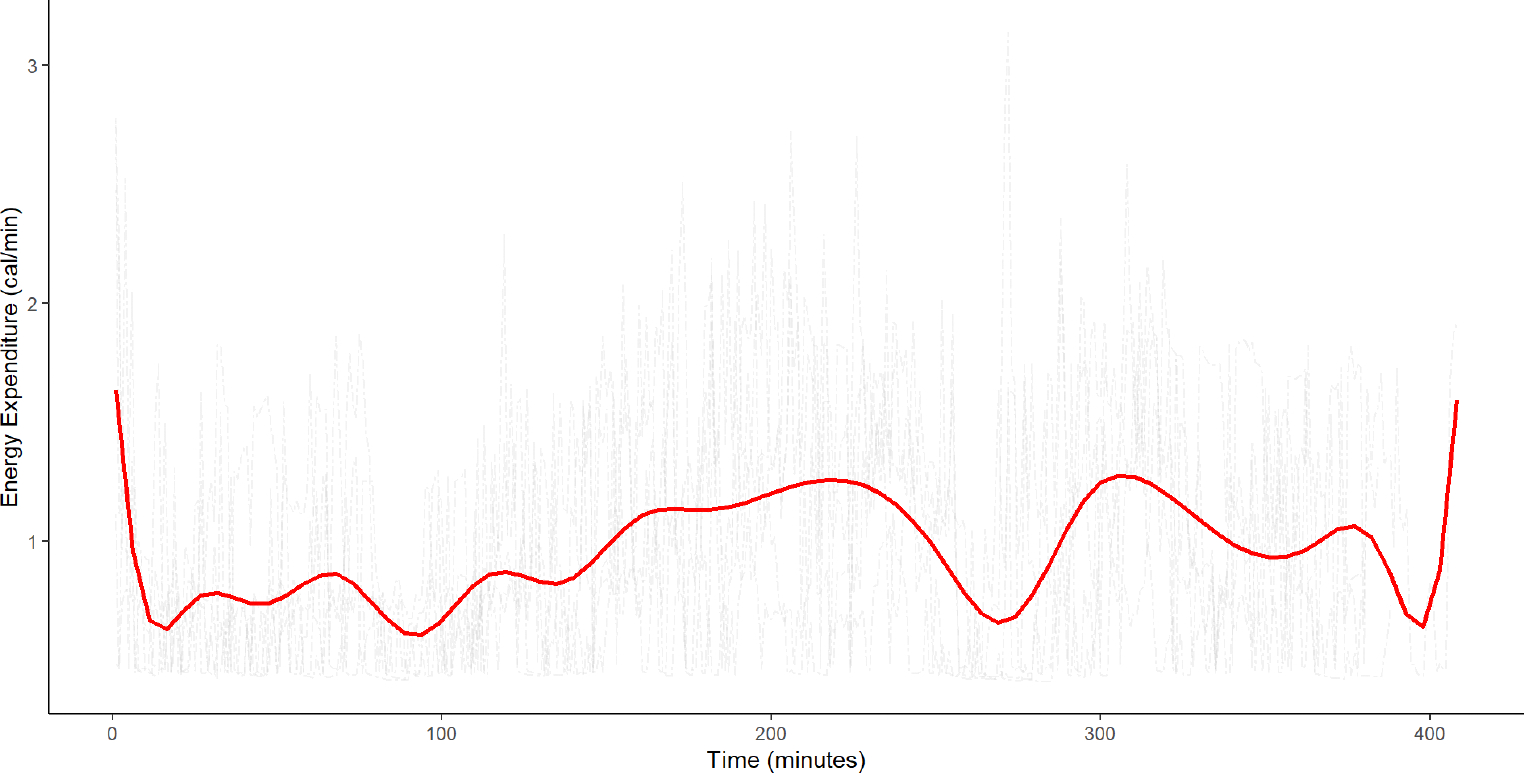
Plot of school-day energy expenditure and mean energy expenditure over 5 days for a randomly selected participant from the stand-biased desk study. The red line represents the smoothed version of the overall mean energy expenditure. The plot illustrates the high-dimensionality of the EE data collected over 5 days.

Using a hierarchical linear mixed effects model, Benden et al. (3) showed that children in stand-biased desk classrooms had significantly higher EE than children in traditional desk classrooms (estimate [Est.] = 0.16, standard error [SE] = 0.04, *p* < 0.001) in the Fall semester, after adjusting for grade, race and gender. However, using a summary value in the hierarchical linear mixed model does not take advantage of the high dimensionality of EE. Figure 1 illustrates such high dimensionality by showing EE data gathered every minute over 5 school days for a randomly selected student participant in our motivating stand-biased desk study. Nonparametric smoothing was used to approximate the average EE recorded over the five school days for a randomly selected student. By smoothing the mean, we uncover underlying patterns in the data while retaining important features (20, 39). The hierarchical linear mixed model does not provide the ability to assess the impact of these underlying patterns on childhood obesity, thus limiting interpretability.

In this manuscript, we use different modeling approaches to examine the relationship between PA and body weight status, as indicated by measures of BMI. We describe the use of a linear mixed-effects model (LMEM), a quantile mixed-effects model (QMEM), a functional mixed-effects model (FMEM), and a functional quantile mixed-effects model (FQMEM) to study the relationship between school-day EE (SDEE) and BMI. We include random effects in all models to account for clustering and treat SDEE as both a scalar-valued summary and a function-valued predictor variable. We discuss the advantages and disadvantages of each modeling approach. The manuscript is organized as follows. In Section 2, we describe the statistical models employed in our applications. The results from our analyses are provided in Section 3, and we offer some concluding remarks in Sections 4 and 5.

## 2 Model specifications

In this manuscript, we analyzed data collected at baseline (Fall 2011). Our analytic sample of 256 participants excluded those with large proportions of missing or incomplete accelerometer data. Mean hourly SDEE values were obtained by calculating hourly averages of minute-level device-measured observations across the 5 days of a school week during which the devices were worn. In our analytic sample, all students had exactly 30 mean hourly SDEE values. For all models, we used log (BMI), the natural logarithm of BMI as the response variable and we adjusted for the following covariates: age, sex (boys vs. girls), and intervention (stand-biased desks vs. traditional desks). Given the cluster-randomized design, we attempted to account for the clustering effects of teachers within schools. However, due to computational and convergence issues, we only included a random intercept for schools in all models. We implemented linear regression and quantile regression models with the R software packages *lme4* (40) and *lqmm* (41, 42), respectively. Below, we provide descriptions of the different models considered.

In the remaining of this section, we provide descriptions of the models considered.

### 2.1 Linear mixed-effects model

Mixed effects models are used to account for clustering in regression models (43). The following model was specified for the LMEM used for students clustered within schools.


$$Y_{ij}=\beta_{0}+\sum_{k=1}^{5}\beta_{1k}X_{ijk}+b_{j}+\varepsilon_{ij},i=1,\ldots ,256$$

In this model, $Y_{ij}=log\left(BMI_{ij}\right)$ represents the response for the ith subject within the jth (j=1,2,3) school, $\beta_{0}$ is a scalar-valued intercept, and $\beta_{1k}$ represents the coefficient on the kth covariate (overall mean SDEE, age, sex, race, and intervention). For SDEE, we obtained the overall mean of the high-dimensional data recorded for each student. We included the random intercept $b_{j}$ to account for clustering within schools, and $\varepsilon_{ij}$ represented the model error associated with $Y_{ij}$. We assume that $b_{j}∼N\left(0,\sigma_{j}^{2}\right)$ and $\varepsilon_{ij}∼N(0,\sigma^{2})$. Random effects and errors terms are commonly assumed to be normally distributed based on large sample theory, and also for mathematical and computational convenience.

### 2.2 Quantile mixed-effects model

The quantile mixed effects model accounts for clustering with random effects in the linear conditional quantile functions (42). We applied the QMEM at the 10th, 25th, 50th, 85th, 95th, and 99th percentiles of the outcome variable, $Y_{ij}=log\left(BMI_{ij}\right)$. Quantile regression models are used to estimate the effects of independent variables on specific quantile levels for a given outcome. We specified the model as follows:

$$Q_{\tau }\left(Y_{ij}\right)=\beta_{0}(\tau )+\sum_{k=1}^{5}\beta_{1k}(\tau )X_{ijk}+b_{j}(\tau ),i=1,\ldots ,256$$

In this model, $Q_{\tau }\left(Y_{ij}\right)$ represents the τth quantile of the outcome for the ith subject within the jth (j=1,2,3) school and is the observed value of the cumulative distribution function of the outcome Y conditional on the covariates. We also define $\beta_{0}(\tau )$ as a scalar-valued intercept for the τth quantile and $\beta_{1k}(\tau )$ as the coefficient on the kth covariate (overall SDEE, age, sex, race, and intervention) for the τth quantile. $b_{j}(\tau )$ accounts for clustering within schools at the τth quantile. SDEE values were obtained by averaging device-based SDEE measures across wear times for each student.

### 2.3 Functional mixed-effects model

The FMEM models fixed and random effects with nonparametric curves (21, 44). In the FMEM, the outcome was $Y_{ij}=log\left(BMI_{ij}\right)$ for the ith subject within the jth school. However, SDEE was treated as a function-valued covariate and modeled as a curve. In general, for the model to be considered an FMEM, the outcome, a predictor, or both must be function-valued. In our application, we employed the model with a scalar-valued outcome and a function-valued covariate. Let $\left(Z_{ij},Y_{ij}\right)$ be a pair of variables, where $Y_{ij}$ is a scalar-valued random variable and $Z_{ij}$ is a random function defined on the unit interval [0,1] such that $Z_{ij}=\left\{Z_{ij}(t),t\epsilon [0,1]\right\}$. The FMEM for the ith subject within the jth (j=1,2,3) school at wear time t is specified as

$$Y_{ij}=\beta_{0}+\int_{0}^{1}\beta_{1}(t)Z_{ij}(t)dt+\sum_{k=1}^{4}\beta_{2k}X_{ijk}+b_{j}+\varepsilon_{ij},i=1,\ldots ,256$$

where $\beta_{0}$ is a scalar-valued intercept, $\beta_{1}(t)$ is a functional coefficient, and $Z_{ij}(t)$ is a function-valued predictor variable. Note that in our application, the wear time t is re-parameterized to the unit interval. The parameter $\beta_{2k}$ represents the coefficient on the kth covariate (age, sex, race, and intervention). The random intercept $b_{j}$ accounts for the clustering within schools, and we assume that $b_{j}∼N\left(0,\sigma_{j}^{2}\right)$ and $\varepsilon_{ij}∼N(0,\sigma^{2})$.

To implement the FMEM, we first represent the functional component with polynomial splines. Then, $\beta_{1}(t)$ becomes $\beta_{1}(t)\approx \sum_{g=1}^{G}\gamma_{g}c_{g}(t)$, where $\gamma_{g}$ are unknown spline coefficients and ${\left\{c_{g}(t)\right\}}_{g=1}^{G}$ are a set of known spline basis functions. The term G indicates the number of basis functions used to approximate the curve associated with $\beta_{1}(t)$, and g indexes the basis functions. The explanatory variable, $Z_{ij}(t)$, can also be expressed as $Z_{ijg}=\int_{0}^{1}Z_{ij}(t)c_{g}(t)dt$. The re-parameterized model becomes

$$Y_{ij}\approx \beta_{0}+\sum_{g=1}^{G}\gamma_{g}Z_{ijg}+\sum_{k=1}^{4}\beta_{2k}X_{ik}+b_{j}+\varepsilon_{ij},i=1,\ldots ,256$$

An advantage of using splines is their flexibility in capturing patterns associated with the functional coefficient, $\beta_{1}(t)$. This model can easily be fitted using both the *lmer* ⁡(40) and *bs* (45) functions in R (46). To assess the effects of SDEE on BMI using the FMEM, we first obtained $\overset{ˆ}{\beta }(t)\approx \sum_{g=1}^{G}{\overset{ˆ}{\gamma }}_{g}c_{g}(t)$. Thus, the initial model containing a function-valued covariate is now re-parameterized to a multivariate linear regression model. However, we note that the new coefficients are not statistically independent. Therefore, although standard packages can be used to estimate the coefficients, estimations of their standard errors must account for correlations among coefficients. To account for these correlations, we employed 95% nonparametric, bootstrap, pointwise, confidence intervals for inferences. For the nonparametric bootstrap, we resampled the original data without replacement. We then estimated the regression coefficients ${\overset{ˆ}{\gamma }}_{g}$, and derived the functional coefficient $\overset{ˆ}{\beta }(t)$ using the resampled data. We repeated the previous steps 1,000 times to obtain 1,000 bootstrap samples with ${\overset{ˆ}{\beta }}_{b}(t),b=1,\ldots ,1000$. Next, we computed the 95% pointwise bootstrap CIs as the 0.025 and 0.975 percentiles of ${\overset{ˆ}{\beta }}_{b}(t)$ at each observed time point (t=1,…,30).

Bootstrap standard errors and p-values were also obtained for the coefficient estimate of each covariate using the function *bootstrap* from the *lmeresampler* package (47).

### 2.4 Functional quantile mixed-effects model

Functional quantile mixed-effects model combines quantile regression with functional data analysis by assuming that regression at different quantiles share some common patterns that can be summarized by a small number of features (48, 49). The FQMEM estimates the effects of predictor variables or interventions on quantile levels of a given outcome while adjusting for clustering. In our application, the model was applied with SDEE as a functional predictor at the 10th, 25th, 50th, 85th, 95th, and 99th percentiles of the outcome variable $Y_{ij}=log\left(BMI_{ij}\right)$. Following the expansion of the functional covariate using polynomial splines, as described in Section 3.3, the reparametrized FQMEM is expressed as

$$Q_{\tau }\left(Y_{ij}\right)\approx \beta_{0}(\tau )+\sum_{g=1}^{G(\tau )}\gamma_{g}(\tau )Z_{ijg}+\sum_{k=1}^{4}\beta_{2k}(\tau )X_{ijk}+\;\;b_{j}(\tau ),i=1,\ldots ,256$$

where $Q_{\tau }\left(Y_{ij}\right)$ represents the τth quantile for the outcome for the ith subject within the jth (j=1,2,3) school, and $\gamma_{g}(\tau )$ is the gth unknown spline coefficients associated with the τth quantile. We also include $\beta_{0}(\tau )$ as a scalar-valued intercept for the τth quantile, $\beta_{1k}(\tau )$ to represent the coefficient on the kth covariate (age, ${se}_{x}$, race, and intervention) for the τth quantile, and $b_{j}(\tau )$ to account for clustering within schools at the τth quantile. The *lqmm* function in R (41, 42) was used to fit the model. Similar to the FMEM, we obtained ${\overset{ˆ}{\beta }}_{\tau }(t)$ from our estimated coefficients ${\overset{ˆ}{\gamma }}_{g}(\tau )$ using the expression ${\overset{ˆ}{\beta }}_{\tau }(t)\approx \sum_{g=1}^{G}{\overset{ˆ}{\gamma }}_{g}(\tau )c_{g}(t)$. Our inferences were also based on 95% bootstrap, pointwise, confidence intervals. We also computed bootstrap standard errors and p-values for each covariate’s coefficient estimate using the function *boot* from the *lqmm* package.

The number of basis functions, G and G(τ), associated with the functional models control the smoothness of the functional covariate (21). Thus, selecting the number of basis functions is a critical step when considering nonparametric approaches for fitting curves. In our applications, we considered 4–7 basis functions for each model. The Akaike information criteria (AIC) were used to select the best-fitting number of basis functions (between 4 and 7) for each FQMEM (50).

## 3 Results

### 3.1 Descriptive statistics

Table 1 provides the descriptive statistics for our analytic sample. The mean BMI was 17.40 kg/m^2^ (standard deviation [sd] = 2.98 kg/m^2^). The study sample included 123 girls (48%) and 176 white students (69%), and the average age at baseline was 7.73 years (sd = 0.74 years). A total of 150 students (59%) were assigned to stand-biased desks, whereas 106 students (41%) were assigned to traditional desks.

### 3.2 Results from the LMEM

We summarized the frequently obtained device-based SDEE measures for each subject into a scalar-valued measure to obtain the overall average SDEE for use in the LMEM. We observed a positive association between the overall mean SDEE and the mean of log (BMI) after adjusting for intervention, race, age, and sex (estimate [Est.] = 0.366, standard error [SE] = 0.023, *p* < 0.001). On average, boys had a lower log (BMI) than girls (Est. = −0.045, SE = 0.014, *p* < 0.001), and students assigned to stand-biased desks had a lower log (BMI) than those assigned to traditional desks (Est. = −0.049, SE = 0.014, *p* < 0.001). Age did not have a statistically significant effect on log (BMI) (Est. = −0.002, SE = 0.010, *p* = 0.819), and white students tended to have a slightly lower log (BMI) than non-white students (Est. = −0.017, SE = 0.015, *p* = 0.259). However, these interpretations apply primarily to students whose BMI values are near the mean BMI value for the entire analyzed sample distribution. Table 2 provides a summary of the LMEM results.

### 3.3 Results from the QMEM

Applying a QMEM provides additional details by allowing interpretations to be made for various quantile levels of a given outcome variable. We performed analyses at the 10th, 25th, 50th, 85th, 95th, and 99th quantile levels of log (BMI). Table 3 shows the results obtained at each quantile level. Across all quantiles, we observed that an increase in overall SDEE was associated with an increase in log (BMI) at each quantile after adjusting for intervention, age, race, and sex (*p* < 0.001 at all quantiles). The use of the QMEM allows for quantile-specific interpretations at each quantile level of the BMI distribution. At the 10th, 25th, 50th, and 99th quantiles, boys had significantly lower log (BMI) values than girls (*p* < 0.001 at the 10th, 25th, and 50th quantiles; *p* = 0.010 at the 99th quantile). Being assigned to stand-biased desks was associated with a significantly lower log (BMI) than being assigned to traditional desks for all quantiles except the 99th quantile (*p* < 0.001 at the 10th, 25th, 50th, and 85th quantiles; *p* = 0.017 at the 95th quantile). Our models suggest that white students had lower log (BMI) values than non-white students, but this statistically significant difference between white and non-white students was only observed at the lower quantile levels (*p* < 0.001 at the 10th quantile; *p* = 0.004 at the 25th quantile; *p* = 0.002 at the 50th quantile). We observed that an increase in age is associated with a slight decrease in log (BMI) for all quantiles except the 95th quantile; however, this association was statistically significant only at the 25th (*p* = 0.037) and 99th quantiles (*p* = 0.026).

### 3.4 Results from the FMEM

Whereas the previous models summarized SDEE into a single scalar value, using splines in functional regression models allows for flexibility by modeling SDEE as a function of time during the school day. We selected the number of basis functions needed for FMEM analyses by fitting several models with varying numbers of basis functions. The obtained AIC values ranged between −365.7 and − 362.4, with the lowest value, −365.7, achieved with seven basis functions. The basis functions were subsequently used as explanatory variables for SDEE when fitting the FMEM.

**FIGURE 2:**
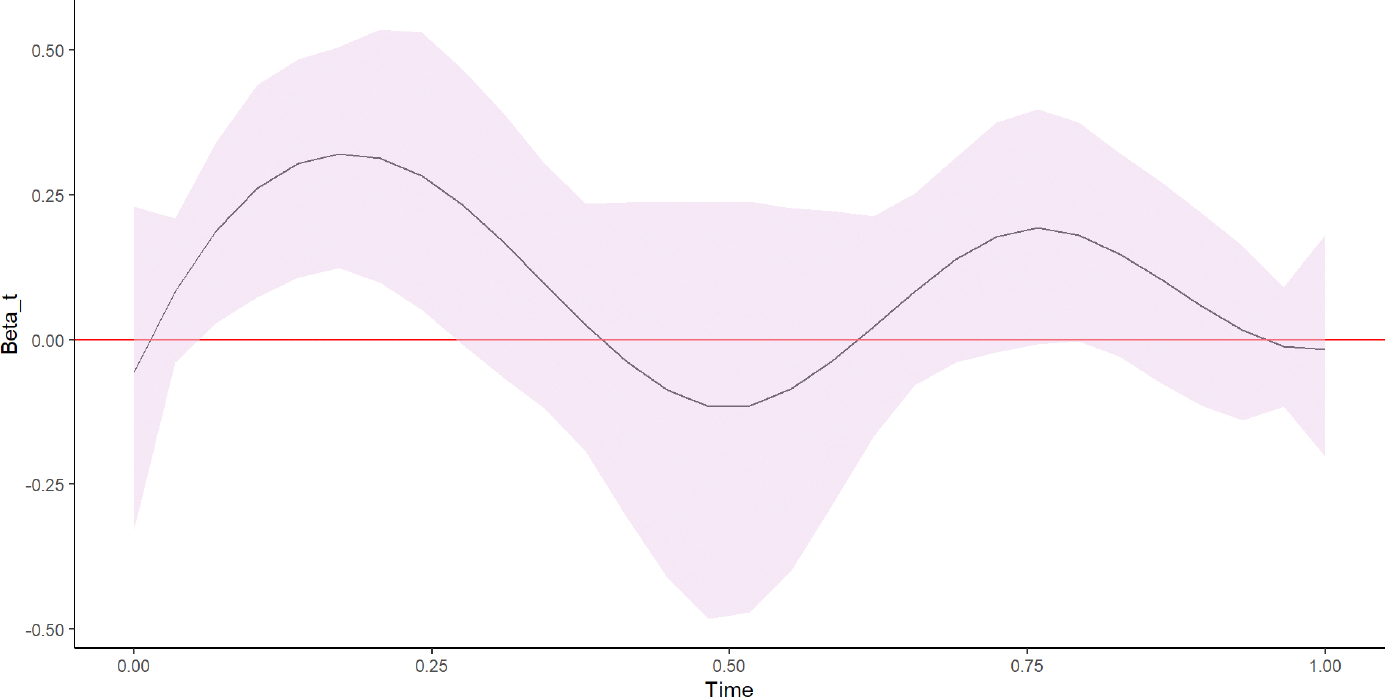
Plot of $\overset{ˆ}{\beta }(t)$ and its 95% bootstrap pointwise confidence interval. The solid black line indicates the estimated functional coefficient on school-day energy expenditure, and the shaded area represents the confidence interval. The curve illustrates that the effect of SDEE on log (BMI) vary across the armband device wear times. The portion of the curve where the zero line is outside the shaded area suggests a statistically significant effect of SDEE on log (BMI) at the corresponding device wear period of time.

Figure 2 shows the estimated functional coefficient, $\overset{ˆ}{\beta }(t)$. The functional coefficient was estimated from a linear combination of the estimated spline coefficients ${\overset{ˆ}{\gamma }}_{g}$ and the basis functions $c_{g}(t)$ using: $\overset{ˆ}{\beta }(t)\approx \sum_{g=1}^{7}{\overset{ˆ}{\gamma }}_{g}c_{g}(t)$. We obtained 95% bootstrap, pointwise confidence intervals.

The estimated functional coefficient illustrates the curvilinear SDEE patterns across periods of device wear time, indicating that PA patterns are not constant across time. Thus, the FMEM provides additional interpretability compared with the LMEM, which uses a scalar-valued SDEE summary as the predictor. In Figure 2, we observe that the horizontal zero line is within the 95% confidence interval bounds. Thus, our results suggest that the FMEM provides insufficient evidence to support a statistically significant effect of SDEE on log (BMI). However, between approximately the 3rd and the 9th hours of wear (t=0.1 to t=0.3), both the upper and lower bounds of the bootstrap confidence intervals are above the zero line, suggesting some effect of SDEE on log (BMI) during this time interval. We also observed that the association between SDEE and log (BMI) depends on the wear time. The results obtained for covariates included in the FMEM were similar to the results obtained for covariates in the LMEM (see Table 4). Boys had lower log (BMI) values than girls, and students assigned to stand-biased desks had lower log (BMI) values than those assigned to traditional desks (*p* < 0.05). No statistically significant association was observed between age and log (BMI) (*p* = 0.476), and white students tended to have slightly lower log (BMI) values than non-white students (*p* = 0.053).

### 3.5 Results from the FQMEM

The flexibility allowed by using splines to study curvilinear SDEE patterns can be further applied to different quantile levels of log (BMI), allowing for interpretations among students with BMI values beyond the mean. At each quantile level, SDEE values were reduced to linear combinations of splines and basis functions. As described for the FMEM, the numbers of basis functions were selected for the FQMEM by comparing the AIC values computed when using 4–7 basis functions at each quantile. The AIC comparisons led to the selection of 6 basis functions at the 10th quantile, 7 basis functions at the 25th and 95th quantiles, 5 basis functions at the 50th and 85th quantiles, and 4 basis functions at the 99th quantile.

**FIGURE 3:**
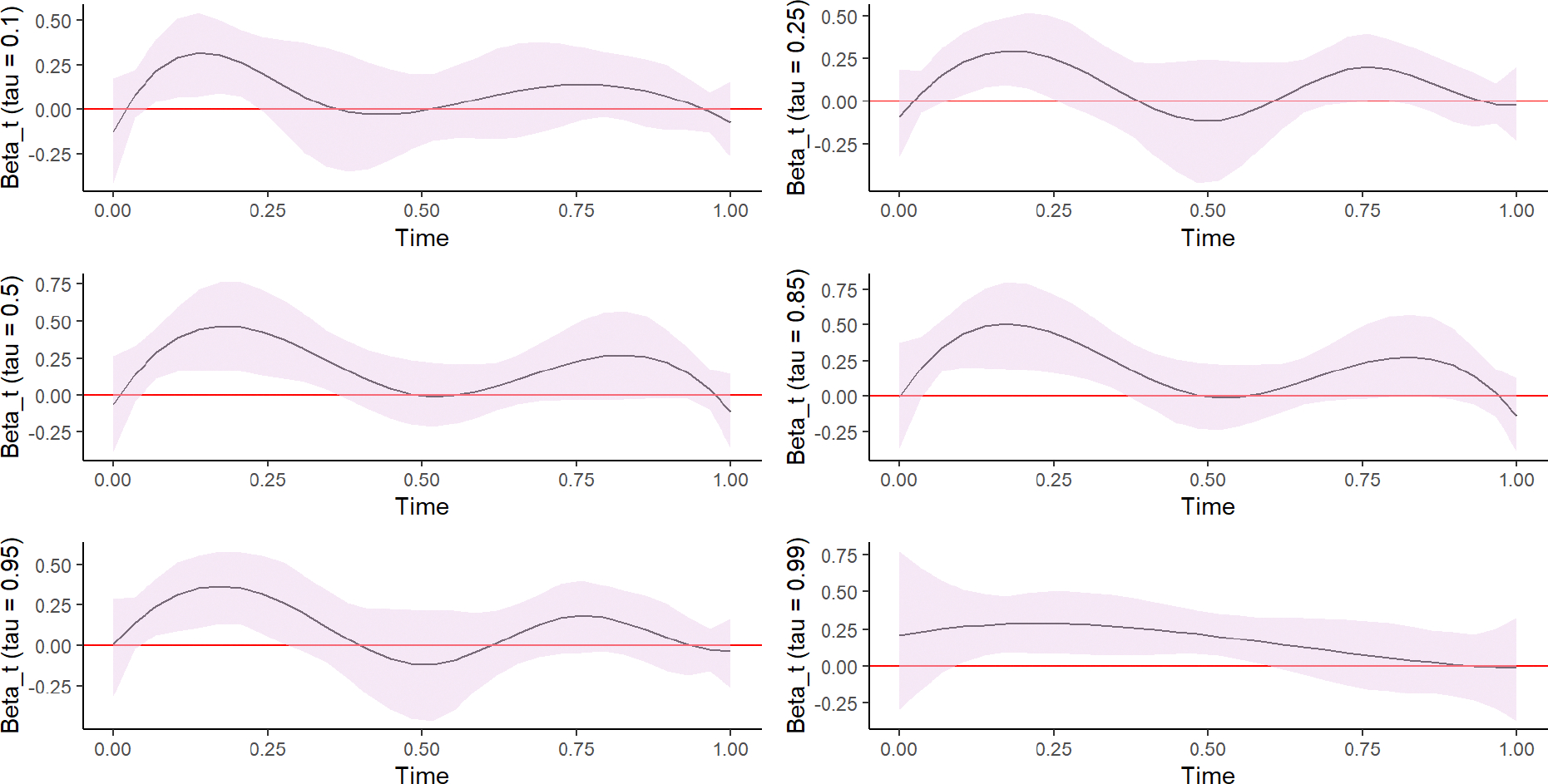
Plot showing the estimated functional coefficients and their corresponding 95% pointwise bootstrap confidence intervals at the 10th, 25th, 50th, 85th, 95th, and 99th quantiles. In each plot, the solid black line indicates the estimated quantile-specific functional coefficient, and the shaded area represents the confidence intervals. The varying patterns across the specified quantiles illustrate that the effect of SDEE differs by the students’ BMI levels. For each curve, the zero line outside the shaded area suggests a significant effect of SDEE on the corresponding log (BMI) quantile, and during a device wear period of time.

Figure 3 provides plots of the estimated effects on functional coefficients on SDEE and their corresponding 95% pointwise confidence intervals. The plots also illustrate SDEE patterns across wear time for each quantile regression. The plots illustrate some statistically significant associations between SDEE and quantile levels of SDEE at certain wear times for the different quantile levels. Specifically, based on the 95% pointwise confidence intervals, a significant effect of SDEE on log (BMI) can be observed for short wear time intervals across all quantiles. These time intervals vary; a significant effect was observed between approximately the 3rd and 7th hours of wear for the 10th quantile and 25th quantiles, the 3rd and 12th hours of wear for the 50th and 85th quantiles, the 3rd and 9th hours of wear for the 95th quantile, and the 3rd and 18th hours of wear for the 99th quantile of log (BMI). In addition, boys had significantly lower log (BMI) values than girls in all assessed quantiles except for the 95th quantile, and students assigned to stand-biased desks had lower log (BMI) values than those assigned to traditional desks across all quantiles (*p* < 0.05, see Table 5). Age did not have a statistically significant effect on log (BMI) (*p* > 0.05 across all quantiles), and white students had significantly lower log (BMI) values than non-white students in the 10th and 50th quantiles only (*p* < 0.001).

## 4 Discussion

We demonstrated and compared mean regression and quantile regression methods for examining the effects of device-based EE measures on BMI. Our analyses indicate that the association between EE and BMI varies depending on how high-dimensional. Device-based EE measures are summarized. When EE measures were summarized into an overall averaged scalar value, we obtained a statistically significant effect of overall mean SDEE on log (BMI). However, when SDEE was considered to be a functional variable, a statistically significant effect of SDEE on log (BMI) was observed for only specific intervals during the device wear time. Thus, spline-based approaches may uncover patterns and allow additional interpretations that are not possible when using approaches that require device-based EE data to be summarized into scalar values. Our results illustrate the complexity of analyzing data collected by devices intended to monitor PA in school-based studies of obesity and body weight status. Despite the existence of much literature describing the obesity-reducing impacts of increasing EE or PA during the school day, we observed that the choice of statistical model is important for accurately assessing the extent of any such relationship.

Across all our models, boys tended to have lower log (BMI) values than girls, and students assigned to stand-biased desks had lower log (BMI) values than those assigned to traditional desks. Age did not have a statistically significant effect on log (BMI), and white students had significantly lower log (BMI) values than non-white students in quantile models at the 10th and 50th quantile levels of log (BMI) only. Based on the 95% pointwise confidence intervals obtained for the FMEM and FQMEM, we observe that the pointwise confidence intervals excluded zero within short time intervals only across the entirety of device wear times, suggesting a significant effect of EE on BMI during these time intervals only and indicated that EE patterns were not independent of time in this study sample. Thus, treating device-measured EE as a function-valued predictor rather than a scalar-valued one may allow for more thorough interpretations of intervention data.

Based on our analyses, no statistical associations between EE and BMI were detected in any of the fitted models. *Ad hoc* comparisons of AICs tended to favor models using a function-valued predictor. For example, the random intercept LMEM produced an AIC of −358.8, whereas the FMEM produced an AIC of −365.7. The small difference in AICs confirmed a slight advantage for models that treat EE as a function-valued predictor when analyzing our data. Similar comparisons were observed when comparing the same quantiles among quantile-based regression models. When comparing across quantiles, AIC values ranged from approximately −400 to 40, illustrating that EE may have differential effects on different BMI categories. Overall, and based on AIC comparisons, regressions at the 25th quantile appeared to provide the best model fit for our analyses.

## 5 Conclusion

We used different regression-based methods to investigate the impacts of EE on BMI among elementary school–aged children recruited from a Texas school district. Using the LMEM, we assessed the effect of overall mean EE on BMI; however, this approach does not account for potential diurnal EE patterns, and the analysis is focused on assessing the conditional BMI mean. Using B-splines in the FMEM uncovered EE patterns and provided more interpretability by modeling objective EE measures as curves. Compared with the spline methodology, methods using the overall mean to represent EE resulted in the loss of information. While both the LMEM and the FMEM enable assessment of covariate effects on the conditional BMI mean, the QMEM and the FQMEM enables assessments of covariate effects across the entire BMI distribution.

One limitation of using quantile regression is related to sample size. Smaller samples tend to limit the implementation of quantile regression models, especially when estimating quantiles of the outcome variable distribution tails or when a large number of covariates are included in the model. Thus, although functional quantile regression models are advantageous for assessing how EE patterns over time affect BMI and enable the effects of these patterns to be assessed across the entire BMI distribution, we recommend the use of functional quantile models for large sample sizes only. For small to moderate sample sizes, estimations around BMI distribution tails may be problematic. Overall, when interest lies in assessing how a function-valued predictor affects children of all body types, quantile regression–based methods are recommended. However, the analytic sample must be sufficiently large to ensure adequate statistical power to assess the effects of PA across the entire BMI distribution.

In our analyses, we accounted for clustering by including random effects for schools using a mixed-effects framework. Failure to account for the cluster-randomized study design may lead to invalid statistical inference, especially for highly clustered data (51, 52). In our analytic sample, the intraclass correlation coefficient was approximately 10%, which highlights the importance of accounting for the clustered effect in our analyses.

## Figures and Tables

**TABLE 1:** Descriptive statistics of the analytic sample (*n* = 256).

	Mean (SD)/*N* (%)
BMI (kg/m^2^)	17.40 (2.98)
Average SDEE (cal/min)	1.32 (0.32)
Age (years)	7.86 (0.80)
Stand-biased desks	150 (58.59%)
Traditional desks	106 (41.41%)
White	176 (68.8%)
Non-white	80 (31.2%)
Boys	133 (51.95%)
Girls	123 (48.05%)

BMI, body max index; SD, standard deviation of the mean; SDEE, school-day energy expenditure.

**TABLE 2:** Results from the linear mixed-effects model.

Fixed effects	Coef. Est.	SE	*P*-value
Intercept	2.391	0.080	<0.001
Average SDEE	0.366	0.023	<0.001
Age	−0.002	0.010	0.819
Boys vs. Girls	−0.045	0.014	<0.001
White vs. Non-white	−0.017	0.015	0.259
Stand-biased vs. Traditional desks	−0.049	0.014	<0.001
Random effects	Var. Est.	SD
School (Intercept)	0.001	0.034
Residual	0.012	0.108

For each student, SDEE is summarized into an averaged scalar value to model the mean of log (BMI). The mixed-effects model includes a random intercept to adjust for the clustering of students within schools.

Coef. Est., coefficient Estimate; SD, standard deviation; SDEE, school-day energy expenditure; SE, standard error; Var. Est., variance estimate.

**TABLE 3:** Results from the quantile mixed-effects models.

τ	Fixed effects	Coef. Est.	SE	*p*-value
0.1	Intercept	2.474	0.070	<0.001
	Average SDEE	0.357	0.022	<0.001
	Age	−0.027	0.014	0.066
	Boys vs. Girls	−0.045	0.010	<0.001
	White vs. Non-white	−0.030	0.008	<0.001
	Stand-biased vs. Control	−0.050	0.006	<0.001
	**Random effects**	**Var. Est.**	**SD**	
	School (Intercept)	1.000		
	Residual	0.016	0.160	
τ	Fixed effects	Coef. Est.	SE	*p*-value
0.25	Intercept	2.475	0.069	<0.001
	Average SDEE	0.357	0.022	<0.001
	Age	−0.020	0.009	0.037
	Boys vs. Girls	−0.044	0.008	<0.001
	White vs. Non-white	−0.028	0.009	0.004
	Stand-biased vs. Control	−0.052	0.005	<0.001
	**Random effects**	**Var. Est.**	**SD**	
	School (Intercept)	1.000		
	Residual	0.031	0.129	
τ	Fixed effects	Coef. Est.	SE	*p*-value
0.5	Intercept	2.476	0.069	<0.001
	Average SDEE	0.361	0.021	<0.001
	Age	−0.013	0.009	0.134
	Boys vs. Girls	−0.043	0.009	<0.001
	White vs. Non-white	−0.030	0.009	0.002
	Stand-biased vs. Control	−0.051	0.005	<0.001
	**Random effects**	**Var. Est.**	**SD**	
	School (Intercept)	1.000		
	Residual	0.042	0.119	
τ	Fixed effects	Coef. Est.	SE	*p*-value
0.85	Intercept	2.522	0.073	<0.001
	Average SDEE	0.393	0.024	<0.001
	Age	0.136	0.070	0.057
	Boys vs. Girls	−0.022	0.013	0.099
	White vs. Non-white	−0.002	0.013	0.849
	Stand-biased vs. Control	−0.063	0.008	<0.001
	**Random effects**	**Var. Est.**	SD.	
	School (Intercept)	0.940		
	Residual	0.039	0.261	
τ	Fixed effects	Coef. Est.	SE	*p*-value
0.95	Intercept	2.511	0.075	<0.001
	Average SDEE	0.371	0.032	<0.001
	Age	0.002	0.136	0.987
	Boys vs. Girls	0.009	0.016	0.583
	White vs. Non-white	0.040	0.029	0.164
	Stand-biased vs. Control	−0.059	0.024	0.017
	**Random effects**	**Var. Est.**	**SD**	
	School (Intercept)	0.974		
	Residual	0.015	0.296	
0.99	**Fixed effects**	**Coef. Est.**	**SE**	**p-value**
	Intercept	2.433	0.065	<0.001
	Average SDEE	0.304	0.026	<0.001
	Age	−0.344	0.150	0.026
	Boys vs. Girls	−0.065	0.024	0.010
	White vs. Non-white	−0.063	0.030	0.041
	Stand-biased vs. Control	−0.034	0.023	0.150
	**Random effects**	**Var. Est.**	**SD**	
	School (Intercept)	0.889		
	Residual	0.008	0.798	

For each student, SDEE is summarized as an averaged scalar value to model specific quantiles of log (BMI). The mixed-effects model includes a random intercept to adjust for the clustering of students within schools. This model allows assessing the effects of SDEE on students with extreme log (BMI) values. τ = 0.1, 0.25, 0.5, 0.85, 0.95 and 0.99 are the quantiles.

Coef. Est., coefficient estimate; SD, standard deviation; SDEE, school-day energy expenditure; SE, standard error; Var. Est., variance estimate.

**TABLE 4:** Results from the functional mixed-effects model.

Fixed effects	Coef. Est.	SE	*p*-value
Intercept	2.407	0.031	0.001
$\overset{ˆ}{\gamma }1$	−0.001	0.335	0.996
$\overset{ˆ}{\gamma }2$	2.112	0.470	0.120
$\overset{ˆ}{\gamma }3$	0.254	0.147	0.261
$\overset{ˆ}{\gamma }4$	−0.288	0.192	0.329
$\overset{ˆ}{\gamma }5$	0.514	0.355	0.266
$\overset{ˆ}{\gamma }6$	0.411	0.632	0.622
$\overset{ˆ}{\gamma }7$	0.031	0.247	0.912
Age	−0.006	0.005	0.476
Boys vs. Girls	−0.049	0.012	0.041
White vs. Non-white	−0.022	0.002	0.053
Stand-biased vs. Traditional desks	−0.050	0.007	0.003
Random effects	Var. Est.		
School (Intercept)	0.001		
Residual	0.011		

SDEE is considered as a curve to model the mean of log (BMI). The model includes a random intercept, which adjusts for the clustering of students within schools. Using SDEE as a curve unmasks some patterns and allow additional interpretation on EE. $\overset{ˆ}{\gamma }1,\ldots ,\overset{ˆ}{\gamma }7$ are estimates for the spline coefficients of SDEE.

Coef. Est., coefficient estimate; SDEE, school-day energy expenditure; SE, bootstrap standard error; Var. Est., variance estimate.

**TABLE 5:** Results from the functional quantile mixed-effects models.

τ	Fixed effects	Coef. Est.	SE	*p*-value
0.1	Intercept	2.480	0.091	<0.001
	$\overset{ˆ}{\gamma }1$	−0.046	0.465	0.921
	$\overset{ˆ}{\gamma }2$	2.136	0.595	<0.001
	$\overset{ˆ}{\gamma }3$	−0.333	0.494	0.500
	$\overset{ˆ}{\gamma }4$	−0.024	0.733	0.974
	$\overset{ˆ}{\gamma }5$	0.692	0.757	0.361
	$\overset{ˆ}{\gamma }6$	0.001	0.358	0.998
	Age	−0.027	0.017	0.118
	Boys vs. Girls	−0.055	0.012	<0.001
	White vs. Non-white	−0.033	0.008	<0.001
	Stand-biased vs. Control	−0.052	0.013	<0.001
	**Random effects**	**Var. Est.**		
	School (Intercept)	1.000		
	Residual	0.016		
τ	Fixed effects	Coef. Est.	SE	*p*-value
0.25	Intercept	2.464	0.082	<0.001
	$\overset{ˆ}{\gamma }1$	−0.008	0.505	0.987
	$\overset{ˆ}{\gamma }2$	1.566	0.865	0.070
	$\overset{ˆ}{\gamma }3$	0.954	0.643	0.138
	$\overset{ˆ}{\gamma }4$	−0.684	0.401	0.089
	$\overset{ˆ}{\gamma }5$	0.667	0.475	0.161
	$\overset{ˆ}{\gamma }6$	0.329	0.597	0.583
	$\overset{ˆ}{\gamma }7$	0.052	0.346	0.880
	Age	−0.019	0.011	0.087
	Boys vs. Girls	−0.054	0.011	<0.001
	White vs. Non-white	−0.012	0.007	0.106
	Stand-biased vs. Control	−0.051	0.034	<0.001
	**Random effects**	**Var. Est.**		
	School (Intercept)	1.000		
	Residual	0.030		
τ	Fixed effects	Coef. Est.	SE	*p*-value
0.5	Intercept	2.488	0.096	<0.001
	$\overset{ˆ}{\gamma }1$	0.161	0.477	0.735
	$\overset{ˆ}{\gamma }2$	2.230	0.982	0.023
	$\overset{ˆ}{\gamma }3$	−2.090	1.148	0.069
	$\overset{ˆ}{\gamma }4$	1.326	0.666	0.047
	$\overset{ˆ}{\gamma }5$	−0.080	0.262	0.760
	Age	−0.016	0.012	0.186
	Boys vs. Girls	−0.046	0.010	<0.001
	White vs. Non-white	−0.034	0.008	<0.001
	Stand-biased vs. Control	−0.054	0.011	<0.001
	**Random effects**	**Var. Est.**		
	School (Intercept)	1.000		
	Residual	0.041		
τ	Fixed effects	Coef. Est.	SE	*p*-value
0.85	Intercept	2.490	0.083	<0.001
	$\overset{ˆ}{\gamma }1$	0.162	0.427	0.705
	$\overset{ˆ}{\gamma }2$	2.231	0.894	0.013
	$\overset{ˆ}{\gamma }3$	−2.089	1.111	0.060
	$\overset{ˆ}{\gamma }4$	1.327	0.676	0.050
	$\overset{ˆ}{\gamma }5$	−0.080	0.253	0.753
	Age	−0.001	0.066	0.993
	Boys vs. Girls	−0.044	0.012	<0.001
	White vs. Non-white	−0.033	0.018	0.072
	Stand-biased vs. Control	−0.055	0.012	<0.001
	**Random effects**	**Var. Est.**		
	School (Intercept)	1.000		
	Residual	0.027		
τ	Fixed effects	Coef. Est.	SE	*p*-value
0.95	Intercept	2.513	0.084	<0.001
	$\overset{ˆ}{\gamma }1$	−0.001	0.506	0.999
	$\overset{ˆ}{\gamma }2$	1.577	0.864	0.068
	$\overset{ˆ}{\gamma }3$	0.968	0.643	0.133
	$\overset{ˆ}{\gamma }4$	−0.669	0.403	0.098
	$\overset{ˆ}{\gamma }5$	0.674	0.475	0.156
	$\overset{ˆ}{\gamma }6$	0.331	0.597	0.580
	$\overset{ˆ}{\gamma }7$	0.053	0.347	0.878
	Age	0.000	0.134	0.998
	Boys vs. Girls	0.008	0.018	0.630
	White vs. Non-white	0.005	0.030	0.878
	Stand-biased vs. Control	−0.053	0.015	<0.001
	**Random effects**	**Var. Est.**		
	School (Intercept)	0.970		
	Residual	0.014		
τ	Fixed effects	Coef. Est.	SE	*p*-value
0.99	Intercept	2.460	0.073	<0.001
	$\overset{ˆ}{\gamma }1$	0.686	0.347	0.048
	$\overset{ˆ}{\gamma }2$	0.844	1.114	0.449
	$\overset{ˆ}{\gamma }3$	−0.486	0.970	0.617
	$\overset{ˆ}{\gamma }4$	0.329	0.233	0.157
	Age	−0.106	0.130	0.415
	Boys vs. Girls	−0.052	0.013	<0.001
	White vs. Non-white	−0.075	0.047	0.113
	Stand-biased vs. Control	−0.057	0.021	0.007
	**Random effects**	**Var. Est.**		
	School (Intercept)	0.932		
	Residual	0.004		

SDEE is considered as a curve to model specific quantiles of log (BMI). A random intercept adjusts for the clustering of students within schools. The model assesses the effects of a curvilinear SDEE on specific quantiles of log (BMI), providing additional interpretations for students with extreme log (BMI) values. τ = 0.1, 0.25, 0.5, 0.85, 0.95 and 0.99 are the quantiles. $\overset{ˆ}{\gamma }1,\ldots ,\overset{ˆ}{\gamma }7$ are estimates for the spline coefficients of SDEE at each quantile.

Coef. Est., coefficient estimate; SDEE, school-day energy expenditure; SE, bootstrap standard error; Var. Est, variance estimate.

## Data Availability

The data analyzed in this study is subject to the following licenses/restrictions: The data set and the R codes for the analyses performed in the present manuscript are available from the corresponding author on request. Requests to access these datasets should be directed to GH, gilson.honvoh@cchmc.org.
